# The Complete Mitochondrial Genome of a Natural Triploid Crucian Carp Mutant, *Carassius auratus* var. *suogu*, and Its Phylogenetic Analysis

**DOI:** 10.3390/life15081156

**Published:** 2025-07-22

**Authors:** Yicheng Zhou, Binhua Deng, Shengyue Lin, Shuzheng Ye, Peng Zheng, Guojun Cai, Weiqian Liang, Chong Han, Qiang Li

**Affiliations:** School of Life Sciences, Guangzhou University, Guangzhou 510006, China; ztc1756822657@e.gzhu.edu.cn (Y.Z.); 32314130055@e.gzhu.edu.cn (B.D.); 32314130041@e.gzhu.edu.cn (S.L.); yeshuzheng@e.gzhu.edu.cn (S.Y.); 32214100085@e.gzhu.edu.cn (P.Z.); 32414100045@e.gzhu.edu.cn (G.C.); 32214130052@e.gzhu.edu.cn (W.L.)

**Keywords:** mitochondrial genome, *C. auratus* var. *suogu*, phylogenetic relationships

## Abstract

*Carassius auratus* var. *suogu*, an endemic fish in southern China, is a natural triploid crucian carp mutant. In this study, the characteristics of mitochondrial DNA sequences were analyzed to understand their taxonomic status and genetic background at the gene level. The complete mitochondrial genome of *C. auratus* var. *suogu* (length, 16,580 bp) comprises 37 genes (13 protein-coding genes, 22 transfer RNA (tRNAs) genes, and 2 ribosomal RNA (rRNAs) genes) and a non-coding control region. The RSCU of the mtDNA of *Carassius* was similar. Ka/Ks analyses showed the ND4 gene had the highest evolutionary rate. Moreover, the whole mitogenome sequences and D-loop region were employed to examine phylogenetic relationships among *C. auratus* var. *suogu* and other closely related species. The result indicated that *Carassius auratus suogu var* clustered with *Carassius auratus auratus* and divided *Carassius* into four clades, providing new insights and data support for the taxonomic status of *Carassius*.

## 1. Introduction

The vertebrate mitochondrial genome is a single extrachromosomal circular DNA molecule of 16–18 kb, generally containing 37 genes: 2 ribosomal RNA genes (rRNAs), 22 transfer RNA genes (tRNAs), and 13 protein-coding genes, as well as non-coding regions [1,2,3]. The gene order is highly conserved among vertebrates, with a few notable exceptions, such as certain amphibians and fish species [4]. In comparison with the nuclear genome, mitochondrial genes are distinguished by their small molecular weight, high copy number, and relative ease of isolation and purification. Contemporary scientific consensus regards mitochondrial DNA (mtDNA) as a highly informative molecular marker that has been widely applied in various fields, including species identification, evolutionary biology, population genetics, and conservation biology [5,6].

Triploid crucian carp is a very common species that can be divided into natural varieties such as *Carassius auratus* ssp. *Pingxiang* [7] and artificial cultivation, such as Xiangyun crucian carp [8]. Triploid fish have the advantages of infertility, fast growth, strong stress resistance, and good meat quality, which has important economic value [9]. *Carassius auratus* var. *suogu* is a naturally triploid crucian carp mutant that is endemic to Guangdong province and Hunan province in China. Only in Guangdong province is there a certain breeding scale [10]. Morphologically, in comparison with other *C. auratus*, the posterior vertebrae of *C. auratus* var. *suogu* begin to atrophy at approximately one-third of the anterior base of the dorsal fin. Additionally, its caudal stalk is significantly shorter than that of other *C. auratus.* The body length is about 1.6 times the body height. Both sides of the dorsal fin posterior base are muscular and slightly raised. The anterior compartment of the swim bladder is much larger than the posterior compartment [11]. The body shape of *C. auratus* var. *suogu* is not a result of deformity or pathology but the product of natural selection throughout the species’ evolutionary history, and it is heritable. Nevertheless, only a limited amount of research has been conducted, mainly focusing on artificial propagation and gene cloning [12,13]. In the prior investigation, no males were identified in the examination of wild stocks, leading to the preliminary conclusion that the *C. auratus* var. *suogu* family lacked a male population and exhibited a gynogenetic reproductive mechanism [14]. A vertebrate with such an exceptional reproductive system is unusual. Further investigations are required to acquire knowledge regarding these freshwater fish species.

To ascertain its genetic background at the gene level, we sequenced and assembled the complete mitochondrial genome of *C. auratus* var. *suogu*, providing insights into its gene content, tRNA structure, and genome architecture in this study. The quantity of protein-coding genes, tRNA genes, base composition, and gene organization in *C. auratus* var. *suogu* were utilized to figure out the evolution and distinctive traits of *C. auratus*. The mitochondrial genome of this fish will facilitate further research on the taxonomy, phylogenetics, and evolutionary biology of this significant species of *Carassius* and species with a close relationship.

## 2. Materials and Methods

### 2.1. Sampling, DNA Extraction, and Polymerase Chain Reaction (PCR)

The *C. auratus* var. *suogu* was obtained from Shaoguan City, Guangdong Province. The fish was subsequently anesthetized and euthanized using MS-222 (STAHERB Co., Ltd., Changsha, China). The tail fin was dissected and kept in 95% ethanol [15] and deposited in the School of Life Sciences, Guangzhou University. Total genomic DNA was extracted from the tail fin samples utilizing the Tissue DNA Kit (TIANGEN Co., Ltd., Beijing, China) according to the manufacturer’s instructions.

The complete mitochondrial genomes of the fishes were amplified using a conventional polymerase chain reaction (PCR). Twenty-one sets of primers were utilized to amplify nearly the entire mitochondrial genome in PCR (Supplementary Table S1). The PCRs were carried out in 25 μL reaction volumes containing 2 × PCR Master Mix (12.5 μL), 1.0 μL of each primer, 2.0 μL of diluted long PCR products, and 8.5 μL ddH_2_O. PCR amplification reactions were performed using the T100 Thermal Cycler (Bio-Rad Laboratories Inc., Shanghai, China) with settings as follows: pre-denaturation at 94 °C for 5 min; 35 cycles of denaturation at 95 °C for 30 s, annealing at 55 °C for 30 s, and extension at 72 °C for 1.5 min. The final extension at 72 °C for 5 min ended the reaction. Purification and sequencing of amplified products were entrusted to Sangon Biotech Company (Shanghai, China).

### 2.2. De Novo Assembly and Annotation of the Mitochondrial Genome

The complete mitogenome was assembled using Bioedit 7.2 [16] with default settings and manually refined if necessary. We assembled the complete mitochondrial genome of *C. auratus* var. *suogu* through comparative analysis of teleost mitochondrial DNA and protein sequences. The map of the mitochondrial genome was visualized using the mitofish online tool [17]. The relative synonymous codon usage (RSCU) of the complete mitochondrial sequence was examined using CodonW 1.4.2 [18]. The nucleotide diversity (Pi) and Ka/Ks ratio were calculated by DnaSP 6.0 [19]. The RSCU, Pi, and Ka/Ks ratios were calculated using the mtDNA sequences of *C. auratus* var. *suogu* and seven other *Carassius*, which were downloaded from NCBI (Supplementary Table S2). The AT skew was calculated under the formula: AT skew = (A − T)/(A + T). The cloverleaf secondary structure of tRNA was deduced based on MITOS and VARNA online tools [20,21,22]. All the statistical charts were plotted by OriginPro2024.

### 2.3. Phylogenetic Analysis

Phylogenetic trees were constructed based on complete mitochondrial genomes and the D-loop region, which were constructed by MEGA 12.0 [23], comprising several *Carassius* species and applying *Cyprinus* as the outgroup. All sequences used for phylogenetic analysis were downloaded from NCBI. Phylogenetic analysis was conducted with the maximum-likelihood (ML) method with a bootstrap value = 1000 [24].

### 2.4. Data Availability

The recently sequenced mitogenome of *C. auratus* var. *suogu* has been submitted to NCBI under GenBank accession number PV631140.

## 3. Results

### 3.1. Genome Content and Organization

**Figure 1:**
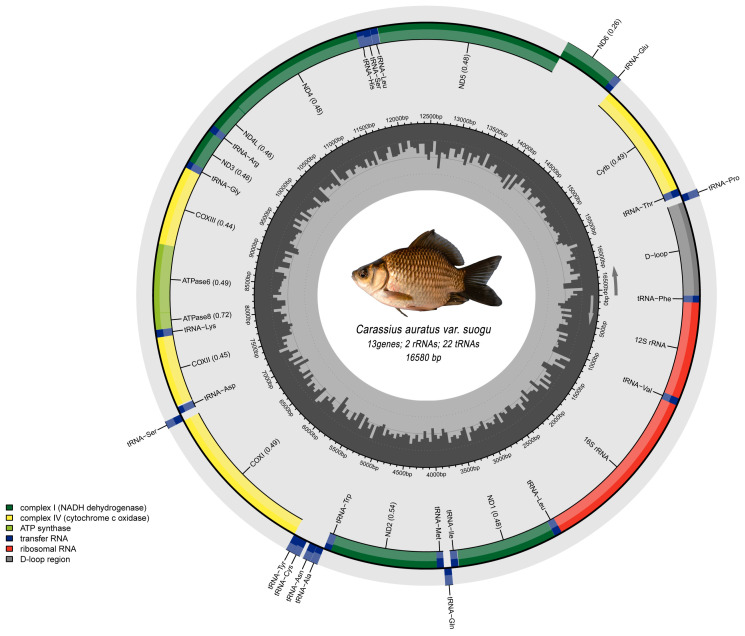
Mitochondrial genome of *Carassius auratus* var. *suogu*. The outer ring represents mitochondrial genes, with the offset ones encoded on the L-strand and the rest on the H-strand.

The complete mitogenome sequence was 16,580 base pairs (bp) in length, including a standard set of 22 tRNAs, 2 rRNAs, 13 typical vertebrate protein-coding genes (PCGs), and a predicted non-coding region (Figure 1). A considerable quantity of genes was encoded on the heavy strand (H-strand). In contrast, eight tRNA genes (tRNA-Gln, tRNA-Ala, tRNA-Asn, tRNA-Cys, tRNA-Tyr, tRNA-Ser (UCN), tRNA-Glu, tRNA-Pro) and *ND6* were located on the light strand (L-strand). Mitochondrial genes overlapped by a total of 22 bp in seven different locations, ranging from 1 bp to 7 bp in length, which were identified among tRNA-Ile, tRNA-Cys, *ATPase 8*, *ND4L*, *ND5*, and tRNA-Thr (Table 1). Additionally, substantial intergenic spacers were identified in the mitochondrial genome of *C. auratus* var. *suogu.* The overall mitochondrial interval was 378 bp across 15 distinct genes.

### 3.2. Protein-Coding Gene (PCGs) and Relative Synonymous Codon Usage (RSCU)

All 13 PCGs recognized in other vertebrates were also discovered in the *C. auratus* var. *suogu*. The total length of PCGs is 11,089 bp, representing 66.8% of the entire mitogenome. Similar to other bony fishes, the mitochondrial genome of *C. auratus* var. *suogu* contains overlapping protein-coding genes. The reading frames of *ATP8-ATP6* and *ND4L-ND4* each exhibited an overlap of seven nucleotides, while the *ND5-ND6* gene pair, encoded on distinct strands, overlapped by four nucleotides (Table 1). In addition, the AT content and AT skew of PCGs of C. auratus var. suogu ranged from 56.62 to 58.46% and 0.025 to 0.048, respectively, which were highly similar to the other three *Carassius* (Table 2).

**Figure 2:**
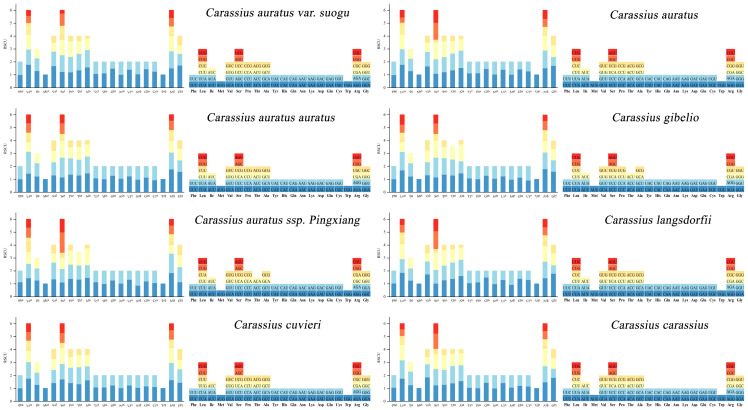
Relative synonymous codon usage (RSCU) of complete mitochondrial sequences of eight species of *Carassius*.

**Figure 3:**
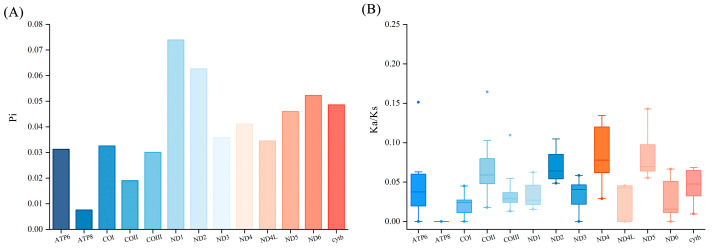
(**A**) Pi and (**B**) Ka/Ks ratio of 13PCGs of eight *Carassius*.

To analyze the codon usage characteristics in Carassius, we calculated the Relative Synonymous Codon Usage (RSCU) for eight Carassius species (Figure 2). The results indicated a similar codon usage pattern among the species. Notably, the codon “ACG” was absent in *C. gibelio* and *C. auratus* ssp. *Pingxiang*. Furthermore, the *ND1* gene exhibited the highest nucleotide diversity (Pi), while *ATP8* displayed the lowest. Additionally, *ND4* had the highest Ka/Ks ratio, whereas *ATP8* had the lowest (Figure 3).

### 3.3. Transfer and Ribosomal RNA Genes, Non-Coding Region

The 12S and 16S rRNA genes were 954 bp and 1682 bp in length, respectively, which total compose 15.89% (2636 bp) of the total mitochondrial genes, and two rRNA molecules were separated by tRNA-Val. Similarly, a pronounced AT-bias was discovered in rRNA, with an average AT content of 55%, while rRNA genes had a high adenine content (Table 1).

**Figure 4:**
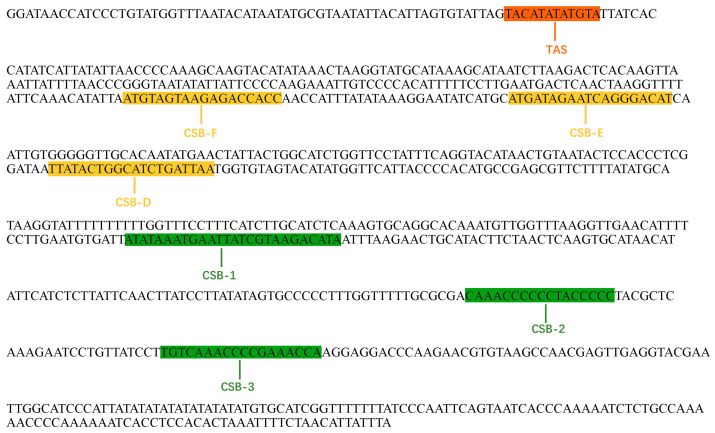
Complete sequences of the control region of *C. auratus* var. *suogu*.

The D-loop measures 924 bp in length, surpassing that of most species. The majority of variation within the fish mitochondrial genome occurs in the D-loop region, which is situated between the tRNA-Pro and tRNA-Phe genes and can be divided into three segments: the terminal associated region (TAS), the central domain (CD), and the conserved sequence blocks (CSB). Key sequences, such as CSB-F, CSB-E, and CSB-D, were identified within the central conserved area. The conserved sequence region found includes CSB-1, CBS-2, and CBS-3, along with additional conserved sequences (Figure 4).

**Figure 5:**
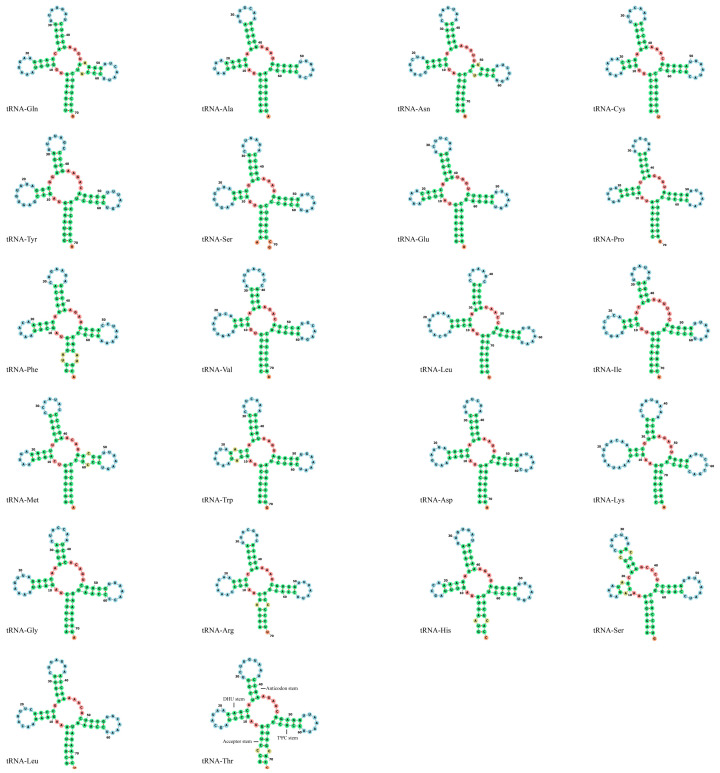
Putative secondary structures of the 22 tRNA genes identified in the mitochondrial genome.

The lengths of 22 tRNA genes of *C. auratus* var. *suogu* vary from 69 bp to 79 bp, comprising 9.43% (1565 bp) of the total mitogenome. The mitochondrial genome comprises 22 typical tRNA genes interspersed among the rRNA and protein-coding genes. All of the tRNA could be folded into a typical cloverleaf secondary structure (Figure 5).

### 3.4. Phylogenetic Analysis

**Figure 6:**
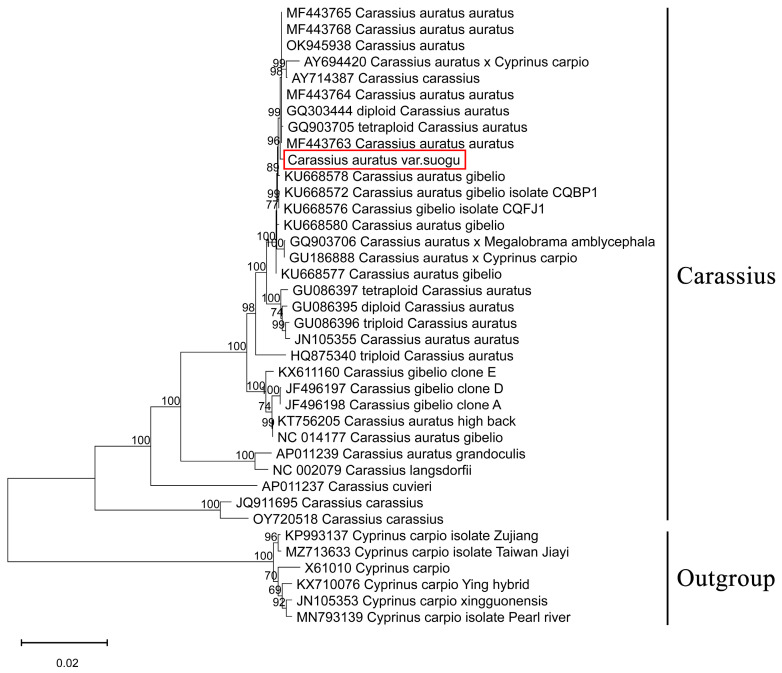
The phylogenetic tree of *C. auratus* var. *suogu* with other *Carassius* based on complete mitochondrial genomes.

**Figure 7:**
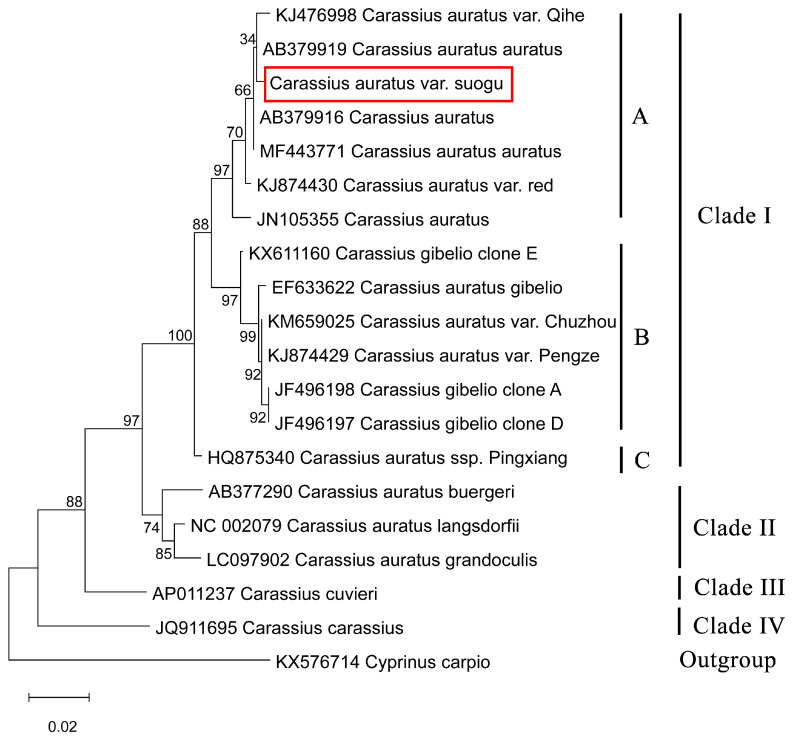
The phylogenetic tree of *C. auratus* var. *suogu* with other *Carassius* based on D-loop region.

The results of phylogenetic trees constructed based on the complete mitochondrial genomes (mitochondrial tree) and D-loop region (D-loop tree) all highly support that Carassius could be divided into four clades. Clade I included *Carassius auratus* (crucian carp), *Carassius auratus auratus* (goldfish), *Carassius auratus gibelio*, and other Chinese *Carassius* varietas. Clade II included *Carassius auratus grandoculis*, *Carassius auratus buergeri*, and *Carassius auratus langsdorfi*. Clade III included *Carassius cuvieri*. Clade IV included *Carassius carassius*. In the D-loop tree, Clade I could be further divided into three groups, which conducted a deeper classification among Chinese *C. auratus*, *C. auratus gibelio*, *C. auratus auratus*, and other Chinese varieties. In addition, *C. auratus* var. *suogu* clustered with *C. auratus* and *C. auratus auratus*, which showed that *C. auratus* var. *suogu* had a closer relationship to *C. auratus* and *C. auratus auratus* compared to other *Carassius* (Figure 6 and Figure 7).

## 4. Discussion

The arrangement of mitochondrial genes serves as a crucial reference for elucidating the evolutionary relationships among species [25]. Comparison of mitochondrial genome arrangements has promise for resolving some of the controversial evolutionary relationships among major animal groups [2]. In this study, we first sequenced the full mitochondrial genome of the endemic triploid fish *C. auratus* var. *suogu*, comprising 37 genes and one D-loop region, as is characteristic of teleost mitogenomes. The mitogenome organization of *C. auratus* var. *suogu* was similar to that of other vertebrates and fish [26,27,28]. Furthermore, we conducted a comparative examination of mitogenome structure, base composition, codon usage, and gene order. The whole mitogenome sequence of *C. auratus* var. *suogu* was 16,580 bp, which was similar to other available sequenced species within *Cyprinidae* [29,30,31,32]. The variance in mitogenome length among these species is primarily attributable to the quantity and size of non-coding regions [21,33]. The high AT content is also observed in the whole mitogenomes, similar to other bony fishes [28].

The characteristics of 13 protein-coding genes are nearly identical to those of other crucian carp [6,34]. Mitochondrial genomes in vertebrates are largely conserved, with a few exceptions noted. The mitochondrial genes of *C. auratus* var. *suogu* exhibit a large base interval between ATPase6 and COIII, and this phenomenon was also found in other species. It was predicted to be relevant to the regulation of mitochondrial gene expression and optimization of mitochondrial function [35,36]. Additionally, the Ka/Ks ratio for *ND4* was significantly raised, indicating a comparatively quick evolutionary pace relative to PCGs. In contrast, the *ATP8* demonstrated the lowest average Ka/Ks ratio, suggesting a reduced evolutionary rate, potentially due to strong selection pressures [37]. Further research is required to elucidate this phenomenon in *C. auratus* var. *suogu*.

We also predicted the 22 tRNAs structure and analyzed the codon usage. All of the tRNA could be folded into a typical cloverleaf secondary structure. Although some studies discovered that many metazoan mitochondrial tRNAs have a loss of Dihydrouracil arms (DHU arms) [38], we did not find such a phenomenon in *C. auratus* var. *suogu*. Investigations of codon bias patterns in genomes can reveal phylogenetic relationships between organisms, horizontal gene transfers, molecular evolution of genes, and identify selective forces that drive their evolution [39]. The codon usage we analyzed in this study could be used to reveal the phylogenetic and evolutionary relationships among different bony fish.

*Carassius* is widely distributed in Asia and Europe. Due to their great viability, complex appearance, multiple ploidy, and reproduction modes, their classification has not been well resolved [40]. However, they can be classified into at least three species: *C. auratus*, *C. carassius*, and *C. cuvieri* [41]. Chinese *auratus* could be further divided into *C. auratus* and C. auratus gibelio morphologically [42]. These perspectives appear to align with ours. Additionally, a previous study based on AFLP analysis has shown that the three Japanese *Carassius* mentioned in this study could be identified as a species different from *C. auratus gibelio* and *C. cuvieri* [43], supporting our findings. However, a research based on SSLP held the view that *C. auratus* and *C. auratus gibelio* are one species [44]. The disparity may arise from significant regional variations in *C. auratus gibelio*.

Human activities have facilitated the movement of local crucian carp species, which were previously isolated and exhibited distinct differences, to various locations, thereby accelerating gene exchange among *Carassius* [29]. The phylogenetic analysis based on complete mitochondrial sequences indicated that *C. auratus* var. *suogu* was clustered with *C. auratus auratus* and *C. auratus red* var., and sister-grouped with *C. auratus gibelo*, corroborating earlier findings derived from previous findings based on the *Cyt-b* gene [45] and complete mitochondrial sequences [46]. Additionally, our result of the phylogenetic tree based on D-loop held a similar view that *C. auratus* var. *suogu* has a close relationship with *C. auratus auratus*. The prior study has demonstrated that *C. auratus auratus* originates from *C. auratus gibelio* [39]. Since *C. auratus* var. *suogu* has a close relationship to C. auratus auratus, it is logical to predict that *C. auratus* var. *suogu* has a similar origin to *C. auratus gibelio.* However, both trees also indicate that *C. auratus* var. *suogu* groups with C. auratus with strong branch support. The precise evolutionary link among them requires further investigation for elucidation.

## 5. Conclusions

This study presents the first sequencing, assembly, and annotation of the mtDNA of *C. auratus* var. *suogu*. The total length of the mtDNA was 16,580 bp, consisting of one non-coding region, 13 protein-coding genes, 22 tRNAs, and 2 rRNAs. In addition, the RSCU of Carassius was similar, and ND1 had the highest Pi, while ND4 had the highest Ka/Ks ratio. Moreover, we examined the phylogenetic relationship of *C. auratus* var. *suogu* and other related species and found that *C. auratus* var. *suogu* has a close relationship with *C. auratus* and *C. auratus auratus*. This study increases the accumulation of experimental data for the subsequent studies of fish and provides new insights into the taxonomic status of *C. auratus* var. *suogu.* Future studies may focus on analyzing the mitogenome of other crucian carps and further explore taxonomic status.

## Figures and Tables

**Table 1:** Gene profile and organization of *C. auratus* var. *suogu* mitochondrial genome.

Gene/Element	Strand	Position	Size (bp)	Start Codon	Stop Codon ^a^	Anticodon	Intergenic Nucleotide ^b^
tRNA-Phe	H	1–69	69			GAA	0
12S rRNA	H	70–1023	954				0
tRNA-Val	H	1024–1095	72			TAC	0
16S rRNA	H	1096–2777	1682				0
tRNA-Leu^(UUR)^	H	2778–2853	76			TAA	+1
*ND1*	H	2855–3829	975	ATG	TAA		+4
tRNA-IIe	H	3834–3905	72			GAT	−2
tRNA-Gln	L	3904–3974	71			TTG	+1
tRNA-Met	H	3976–4044	69			CAT	+10
*ND2*	H	4055–5090	1036	ACG	T--		0
tRNA-Trp	H	5091–5161	71			TCA	+2
tRNA-Ala	L	5164–5232	69			TGC	+1
tRNA-Asn	L	5234–5306	73			GTT	+33
tRNA-Cys	L	5340–5408	69			GCA	1
tRNA-Tyr	L	5408–5478	71			GTA	+1
*COI*	H	5480–7030	1551	GTG	TAA		0
tRNA-Ser^(UCN)^	L	7031–7101	71			TGA	+3
tRNA-Asp	H	7105–7176	72			GTC	+2
*COII*	H	7189–7879	691	ATG	T--		0
tRNA-Lys	H	7880–7955	76			TTT	+1
*ATPase 8*	H	7957–8121	165	ATG	TAG		−7
*ATPase 6*	H	8115–8486	372	ATG	TAG		+310
*COIII*	H	8797–9581	785	ATG	TA-		0
tRNA-Gly	H	9582–9653	72			TCC	0
*ND3*	H	9654–10,002	349	ATG	T--		0
tRNA-Arg	H	10,003–10,072	70			TCG	0
*ND4L*	H	10,073–10,369	297	ATG	TAA		−7
*ND4*	H	10,363–11,743	1381	ATG	T--		0
tRNA-His	H	11,744–11,812	69			GTG	0
tRNA-Ser^(AGY)^	H	11,813–11,881	69			GCT	+1
tRNA-Leu^(CUN)^	H	11,883–11,955	73			TAG	+3
*ND5*	H	11,959–13,782	1824	ATG	TAA		−4
*ND6*	L	13,779–14,300	522	ATG	TAG		0
tRNA-Glu	L	14,301–14,369	69			TTC	+5
*Cyt-b*	H	14,375–15,515	1141	ATG	T--		0
tRNA-Thr	H	15,516–15,587	72			TGT	−1
tRNA-Pro	L	15,587–15,656	70			TGG	0
D-loop	H	15,657–16,580	924				0

^a^ TA- and T-- represent incomplete stop codons. ^b^ Numbers correspond to the nucleotides separating adjacent genes (negative numbers indicate overlapping nucleotides, positive numbers indicate nucleotide intervals).

**Table 2:** AT content and AT skew of PCGs of four Carassius.

Gene	AT Content (%)/AT Skew of CDS							
	*C. auratus* var. *suogu*		*C. auratus*		*C. auratus auratus*		*C. gibelio*	
*ND1*	58.46	0.039	58.55	0.038	58.48	0.039	58.48	0.039
*ND2*	57.80	0.047	57.89	0.046	57.82	0.047	57.82	0.047
*COI*	56.80	0.023	56.76	0.024	56.81	0.023	56.81	0.023
*COII*	57.31	0.032	57.42	0.031	57.35	0.032	57.35	0.032
*ATP8*	58.18	0.048	58.18	0.047	58.18	0.048	58.18	0.048
*ATP6*	57.46	0.035	57.53	0.034	57.49	0.035	57.49	0.035
*COIII*	56.94	0.028	56.89	0.027	56.91	0.028	56.91	0.028
*ND3*	58.17	0.041	58.22	0.040	58.20	0.041	58.20	0.041
*ND4L*	57.89	0.033	57.97	0.032	57.93	0.033	57.93	0.033
*ND4*	57.06	0.029	57.12	0.028	57.09	0.029	57.09	0.029
*ND5*	57.24	0.031	57.31	0.030	57.27	0.031	57.27	0.031
*ND6*	58.24	0.043	58.31	0.042	58.28	0.043	58.28	0.043
*cytb*	56.62	0.025	56.68	0.026	56.65	0.025	56.65	0.025

## Data Availability

The original contributions presented in the study are included in the article; further inquiries can be directed to the corresponding authors.

## Supplementary Materials

The following supporting information can be downloaded at: https://www.mdpi.com/article/10.3390/life15081156/s1, Table S1: Primers used for PCR amplification; Table S2: mtDNA sequences for AT skew, RSCU, and Pi and Ka/Ks ratio.
