# Supplementary material for: The Complete Mitochondrial Genome of a Natural Triploid Crucian Carp Mutant, Carassius auratus var. suogu, and Its Phylogenetic Analysis
Source: Life (Basel). 2025 Jul 22;15(8):1156. doi: 10.3390/life15081156 (PMC12387767; doi:10.3390/life15081156)
Supplement: Supplementary file 1 [file life-15-01156-s001.zip › life-3671371-supplementary.pdf]

Supplementary Table S1. Primers used for PCR amplification

Prime name	Sequence	Length of products
L-prime 1	AGCCAACGAGTTGAGGTACG	984 *bp*
R-prime 1	TGTTTCATGGTGCATGTTCGT	
L-prime 2	CCCCTAGAGGAGCCTGTTCT	984 *bp*
R-prime 2	CTTTGTCAAAGGGGCTGTACC	
L-prime 3	AAACCCCGAAACCAGGTGAG	982 *bp*
R-prime 3	TGGTCGCCCCAACCGAAG	
L-prime 4	CGTGCAAAGGTAGCGCAATC	979 *bp*
R-prime 4	GAGGATGTAGATGGGCGGAC	
L-prime 5	TCCCAGTTTATGCTAAACACCCT	1000 *bp*
R-prime 5	AGGAACTGAGGGGATTTTAACCC	
L-prime 6	TCCTATGAGTACGAGCCTCG	1094 *bp*
R-prime 6	TGTTGTCGTTAGGATGGGGC	
L-prime 7	TTCTAGCCTACTCCTCAATTGC	997 *bp*
R-prime 7	GCGGTTCCTACTATTCCGGC	
L-prime 8	GAAAAGCCCCGGCAGAGTAT	998 *bp*
R-prime 8	GAACCCTAGGAGGCCAATGG	
L-prime 9	ACCCAATTCTCTATCAACACTTATTC	1000 *bp*
R-prime 9	AACCTACAATTTCACCTTGACAAG	
L-prime 10	AGCCTTCGCCGCTAAACG	987 *bp*
R-prime 10	TCCTAGCGAGGCGTCTTCTA	
L-prime 11	CAGTCCCAGGCCGACTAAAT	992 *bp*
R-prime 11	ACTGTGGCGGTTAGAATTGC	
L-prime 12	AGGTACACCCATCCCACTGA	1023 *bp*
R-prime 12	GACAAAGTGTCAGTATCAGGCA	
L-prime 13	GGACTCTACTTTACTGCTCTCCA	1045 *bp*
R-prime 13	GTTTTGTAGTCGGTCAGTGCC	
L-prime 14	GGCCCTATGAGCCCTACAAT	1055 *bp*
R-prime 14	GCTACAAGGCCCATGTGACT	
L-prime 15	AGCAGCAGTCTTACTAAAGCT	1037 *bp*
R-prime 15	AGAATGCGGAACTAACGGCA	
L-prime 16	ATCCAAGTAGAAGCTAAAATGACATT	1085 *bp*
R-prime 16	TGTTCGTCGTTTAGGCTGTGA	
L-prime 17	ACCCAAAACGATATCAAGAAAATTGT	998 *bp*
R-prime 17	ACCCTGCTTGTTGTGTTGGA	
L-prime 18	GCCATAGGACCAAAAGGCCT	1035 *bp*
R-prime 18	CCTCGGGCAATGTGTATGTA	
L-prime 19	CACCTCAGACATTTCAACCGC	981 *bp*
R-prime 19	GGGCAAGCTCATTTCAGTGC	
L-prime 20	CGCAGACATGATTATCCTGACA	990 *bp*
R-prime 20	TGTCGCGCAAAAACCAAAGG	
L-prime 21	AGGTTGAACATTTTCCTTGAATGTGA	980 *bp*
R-prime 21	CCGTCAGGTCCTTTGGGTTT	

Supplementary Table S2. mtDNA sequences for AT skew, RSCU, and Pi and Ka/Ks ratio

Species	Accession Number
Carassius cuvieri	AP011237
Carassius carassius	JQ911695
Carassius auratus	KJ874428
Carassius auratus auratus	MF443764
Carassius langsdorfii	NC_002079
Carassius auratus ssp. Pingxiang	NC_015142
Carassius gibelio	KU668577
